# Supplementary material for: His unemployment, her response, and the moderating role of welfare policies in European countries
Source: PLoS One. 2023 Nov 28;18(11):e0292749. doi: 10.1371/journal.pone.0292749 (PMC10683995; doi:10.1371/journal.pone.0292749)
Supplement: S1 Table — (DOCX) [file pone.0292749.s001.docx]

**S1 Table. Hypothetical Household characteristics for calculating income replacement rates**

| Variables | HH1 | HH2 |
| --- | --- | --- |
| Without children | - | - |
| Age first child | Range: 0-18 Level: 3 | Range: 0-18 Level: 3 |
| Age second child | Range: 0-18  Level: 3 | Range: 0-18 Level: 3 |
| Age third child | Range: 0-18  Level: 3 | Range: 0-18 Level: 3 |
| Union type | Married  Cohabiting | Married  Cohabiting |
| Activity partner | Not working | Employed part-time |
| Transition | T0:Employed  (part-time or full-time)  T1: Unemployed | T0:Employed  (part-time or full-time)  T1: Unemployed |
